# Monitoring migrant groups as a post-validation surveillance approach to contain the potential reemergence of lymphatic filariasis in Togo

**DOI:** 10.1186/s13071-021-04644-2

**Published:** 2021-03-02

**Authors:** Monique Ameyo Dorkenoo, Martin Kouame Tchankoni, Degninou Yehadji, Kossi Yakpa, Mawèké Tchalim, Efoe Sossou, Rachel Bronzan, Didier Koumavi Ekouevi

**Affiliations:** 1 Université de Lomé, Faculté des Sciences de la Santé, BP 1515 Lomé, Togo; 2 Programme National d’Elimination de la Filariose Lymphatique, Ministère de la Santé et de l’Hygiène Publique, BP 336 Lomé, Togo; 3 Division des Laboratoires, Ministère de la Santé et de l’Hygiène Publique, 374 avenue Georges Pompidou, BP 1161 Lomé, Togo; 4 Centre Africain de Recherche en Epidémiologie et en Santé Publique (CARESP), Lomé, Togo; 5 Laboratoire de Parasitologie, Programme National de Lutte contre le Paludisme, Ministère de la Santé et de l’Hygiène Publique, Rue Adamé, Quartier administratif BP 518, Lomé, Togo; 6 Health and Development International (HDI), Newburyport, MA USA

**Keywords:** Lymphatic filariasis, Post-validation surveillance, Migrants group, Togo

## Abstract

**Background:**

In March 2017, Togo was declared the first country in sub-Saharan Africa to eliminate lymphatic filariasis as a public health problem, but post-validation surveillance has been lacking. In some areas of the country, migrant groups from neighboring countries that are still endemic for LF pose a risk of reintroduction of LF to Togo. The objective of this study was to identify the risk posed by migrant groups by measuring their prevalence of LF infection and investigating any positive case using Togo’s case investigation algorithm to prevent resurgence of LF and sustain Togo’s elimination success.

**Method:**

A cross-sectional study was conducted in 2018 in the northernmost region of the country. Three migrant populations were identified: (i) nomadic Peuhls, (ii) Togolese members of local communities who migrate annually to neighboring countries for seasonal labor, and (iii) refugees from Ghana who came to Togo because of a communal conflict in Ghana. A questionnaire was designed to collect data on demographics and history of LF and MDA; all participants were tested for circulating filariasis antigen (CFA) using the filariasis test strip (FTS). Any CFA-positive case was confirmed with nocturnal microfilaremia.

**Results:**

Refugees, seasonal economic migrants and nomadic Peuhls represented 42.1%, 31.4% and 26.5% of the study participants, respectively. The overall prevalence of CFA was 4.2% (58/1391) with the highest prevalence in the nomadic Peuhl group (11.9%), but only one of them (0.07%) was confirmed positive with nocturnal microfilaremia. Using the case investigation algorithm, no other positive case was identified in the positive case’s surroundings.

**Conclusion:**

This study demonstrates that nomadic Peuhls, with a CFA prevalence of 11.9%, pose a potential risk for reintroduction of LF into Togo while Ghanaian refugees and seasonal economic migrants do not appear to pose a significant risk. Periodic monitoring of migrants, especially the nomadic Peuhl population, is a potential post-validation surveillance approach that could be used to promptly detect any LF cluster that may arise.

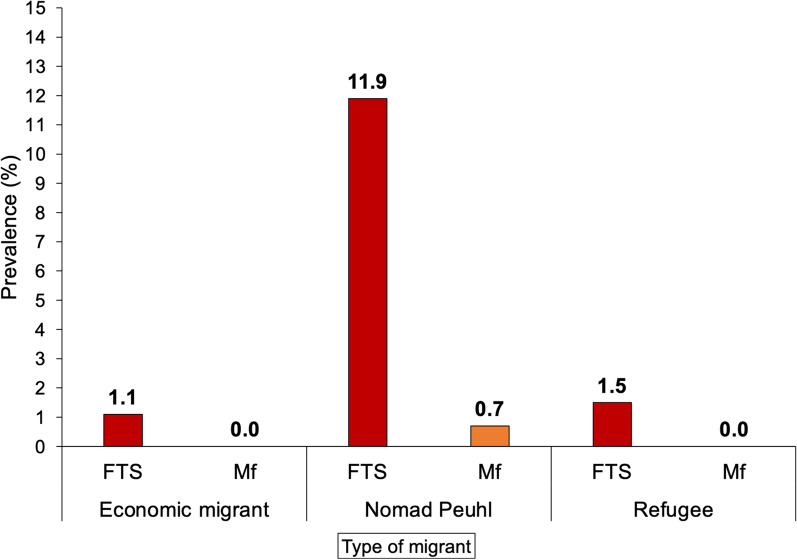

## Introduction

Lymphatic filariasis (LF) is a parasitic infection that is spread by mosquitos infected with worm larvae. LF is the second most common vector-borne parasitic disease after malaria and is endemic in over 83 tropical and subtropical countries [1]. In sub-Saharan Africa, LF is caused by the parasitic nematode *Wuchereria bancrofti* and transmitted by Anopheles, Culex, and Mansonia mosquitoes [2]. To interrupt LF transmission, an annual single dose of albendazole is given in combination with diethylcarbamazine or ivermectin to LF-endemic communities through mass drug administration (MDA) [1, 3]. As of October 2018, 49 countries remain that require preventive chemotherapy for LF. The World Health Organization (WHO) has formally validated the elimination of LF as a public health problem in 16 countries and territories in the world, including Togo [4, 5].

In 2000, LF disease mapping found 8 of the 40 districts of Togo to be endemic for LF; from 2000 to 2009, MDA with albendazole plus ivermectin was implemented in these districts with satisfactory performance indicators. Two transmission assessment surveys (TAS) were conducted in Togo in 2012 and 2015, 3 and 6 years, respectively, after MDA was discontinued and according to WHO recommendations. At the same time, a passive surveillance system based on the laboratory network covering the entire country was implemented from 2006 to 2015 to detect any new active foci of LF [5, 6]. This post-treatment surveillance identified only sporadic infections and no evidence of ongoing transmission of the parasite across the territory. In light of these favorable outcomes, in March 2016, Togo applied for validation of the elimination of LF as a public health problem, and in 2017 Togo became the first country in sub-Saharan Africa to receive WHO validation of the elimination of LF as a public health problem [7, 8]. Although MDA specific for LF ceased in 2010, Togo continues MDA with albendazole in school-age children for soil-transmitted helminths (STH) and ivermectin for onchocerciasis where both diseases are still endemic.

WHO also recommends establishing post-validation surveillance, but without clear guidelines on how this should be done. Therefore, several alternative strategies have been tested through operational research projects in Togo. In 2016, all positive cases identified during the post-treatment surveillance activities conducted from 2006 to 2015 were traced to follow-up on their infection status, and, in 2017, the mosquito vectors were assessed to determine the presence of LF infection. The results of those recent studies confirmed the absence of ongoing LF transmission in Togo [9].

In 2014, health center managers in the Savanes Region of Togo (the northernmost region of the country) reported the presence of migrant groups entering from neighboring countries that are still endemic for LF. Migrants who arrive and reside temporarily but recurrently in some localities of this region could be a source of resurgence of disease, particularly since the nationwide passive surveillance stopped in 2016.

In order to track all population movements, especially migrant groups that constitute a potential risk of the reemergence of LF in Togo, this study was conducted to describe the migrant groups in the northern districts in Togo, to determine LF transmission status among these migrant groups, and to investigate any positive cases following the algorithm of Togo’s passive surveillance system.

## Methods

### Study design

**Fig. 1:**
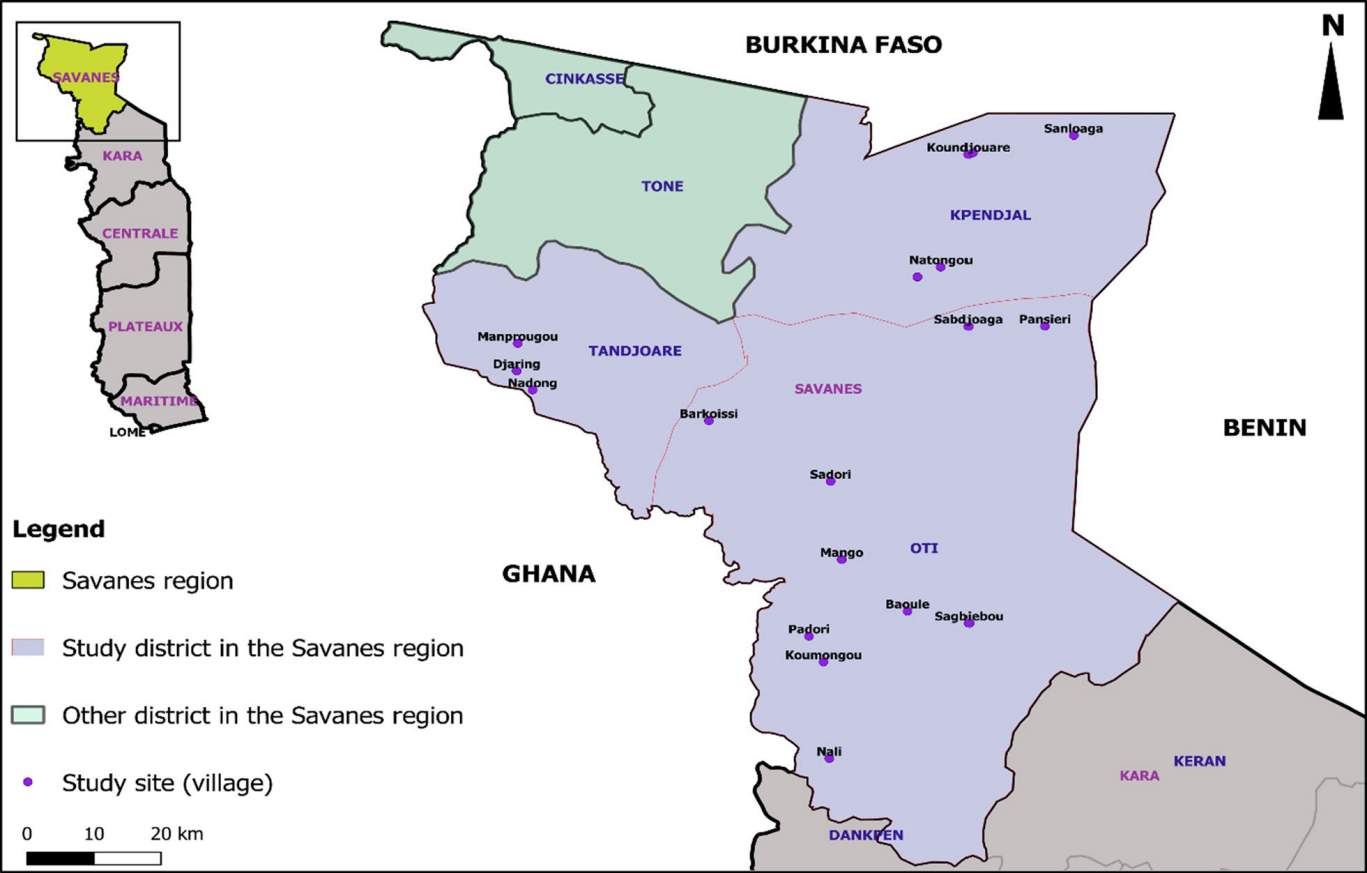
Togo map showing the study region, districts, and sites

This was a cross-sectional study conducted from January to March, 2018 in Savanes region, the northernmost region of Togo, which is subject to regularly recurring migrant movements. The study defined a migrant as a non-resident person passing through or temporarily residing in the locality of the study area. The Savanes region is split into five districts: Oti, Tandjouare, Kpendjal, Cinkassé, and Tone (Fig. 1), of which the last three districts (Kpendjal, Cinkassé, and Tone) were endemic to LF. The Savanes Region is adjacent to Burkina Faso (the neighboring country in the northern part of Togo) where LF transmission is still sustained despite many rounds of MDA [9]. Moreover, during the TAS implemented in 2012 and 2015, the majority of positive cases were found in these three districts of the Savanes region [6, 10].

The study period was determined based on (i) the migratory calendar of the migrant groups: the Peuhls, who cross the territory between December and May coming from Burkina Faso to Ghana, and the Togolese migrating to neighboring countries for seasonal labor, who return to Togo between December and May, and (ii) the reliability of biological results; the assessment should be conducted at least 6 months after the end of the integrated MDA with albendazole, ivermectin, and praziquantel for STH, onchocerciasis, and schistosomiasis, conducted from August 19 to September 7, 2017.

### Study population, sample size, and inclusion criteria

Ten migrant sites were identified for the assessment, and, based on a minimum number of 100 persons targeted for enrollment at each site and a non-response rate estimated at 15% [10, 11], the sample size was estimated at 1150 persons. The three groups defined as migrants in the study area were: (i) nomadic Peuhls who cross Kpendjal and Oti districts, part of the transhumance corridor; (ii) members of local communities in the districts of Kpendjal and Tandjouare, who migrate annually to neighboring countries (Ghana, Benin, and Burkina Faso among others) for seasonal labor in May and return to Togo in December; (iii) residents of refugee camps in the district of Tandjouare, who arrived in the district because of a communal conflict in Ghana and were hosted by inhabitants of the same ethnic group who live in Togo. As these migrants have mixed with local communities, all households and neighborhoods hosting a migrant have been included. Any member of these three migrant groups who consented (or whose parents consented) to participate was included.

No migrant groups were identified in Tone or Kpendjal district, the two remaining districts of the Savanes region.

### Testing for *W. bancrofti* circulating antigen

The filariasis test strip (FTS) (Alere Inc., Waltham, MA, USA), which detects *W. bancrofti* antigens, was used to test study participants. Capillary blood collected at the fingertip of each of participant was applied to the test strip following the test kit operating procedures. If the FTS result was positive, it was repeated, as is done for Togo’s protocol developed for passive LF surveillance. For cases where the repeat test was also positive, the FTS result was considered positive; if the second was negative, a third test was done to establish the final result. Only two (3%) samples that were positive on the first FTS ultimately required a tie-breaker third test. Positive and negative controls were used to validate the tests before their use. According to the passive surveillance algorithm developed by the Togo Ministry of Health [6], nocturnal microfilaremia was performed for all FTS positive cases. The nocturnal thick blood smear was carried out between 10:00 p.m. and 3:00 a.m., using one drop (~ 50 µl) of finger stick blood, to search *W. bancrofti* microfilariae. Giemsa stain diluted at 3% was used for all blood slides, which were conveyed to the reference laboratory of the National Program for elimination of LF (NPELF) for microscopic examination for quality control, where a qualified microscope operator independently read all of the slides.

### Epidemiological investigation of microfilaremia and FTS-positive cases

According to Togo’s algorithm for passive surveillance, established in 2006 [6], a case investigation was immediately launched for any person with two positive FTS results (regardless of that person’s eventual results of the nocturnal smear) by screening all family members and all residents of the other households within the compound for *W. bancrofti* microfilaremia using nocturnal smears [5, 6, 11].

### Data collection and analysis

A dedicated questionnaire was administrated to enrolled participants to collect sociodemographic information, their place of origin, duration of stay in Togo, history of bednet use and other LF prevention strategies, and the number of LF MDA rounds previously received in Togo or from their country of origin. The data were collected on a printed form and entered and cleaned in a Microsoft® Access (Redmond, WA, USA) database.

Frequencies were compared using Chi-square test or Fisher exact test. All statistical tests were performed at the 5% level of significance. All analyses were performed using R® Software (R Foundation for Statistical Computing, Vienna, Austria).

### Ethical considerations and human subject protection

The protocol for this study was approved by the Bioethics Committee for Health Research of the Togo Ministry of Health (approval no. 390/2017/MSPS/CAB/SG/DGAS/DPML/CBRS). In addition, written informed consent was obtained from every adult enrolled and from the parent or guardian of each child enrolled in this study. Data collected are kept secured with a password. Only the investigation team has access to the data. No personal identification information has been reported. All persons testing positive by FTS and/or microfilaremia received a single dose of ivermectin plus albendazole according to WHO MDA dosing.

## Results

### Sociodemographic and clinical characteristics

**Fig. 2:**
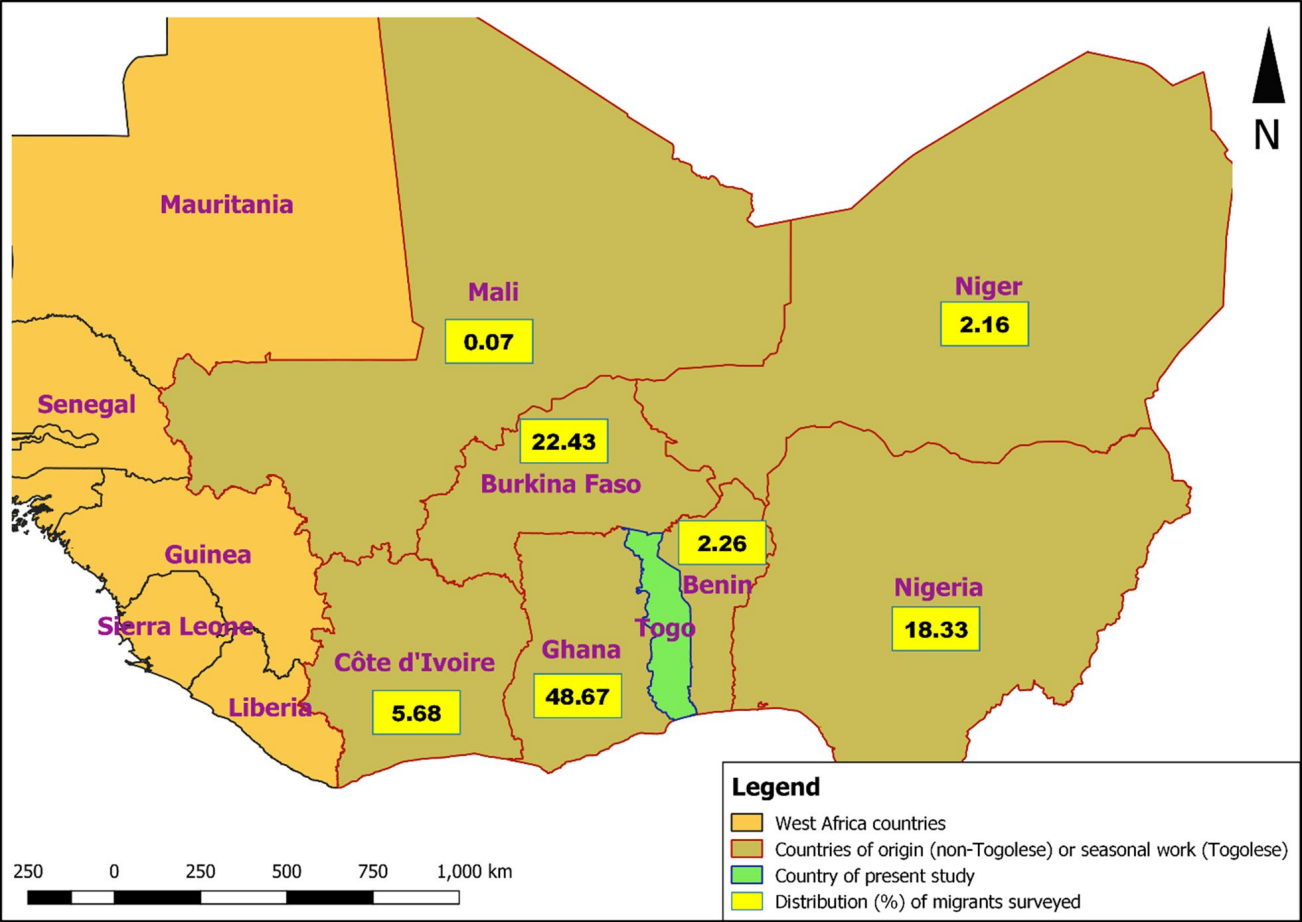
Distribution (%) of surveyed migrants by country of origin (for refugees and Peuhls) or site of seasonal work (for Togolese)

A total of 1391 people including 700 women (50.3%) were enrolled and tested in three health districts (Kpendjal, Oti, and Tandjouare). Around half of the participants (46.6%) were surveyed in Tandjouare, 32.6% in Oti, and 20.8% in Kpendjal. Seasonal economic migrants, nomadic Peuhls, and refugees represented 31.4%, 26.5%, and 42.1% of the study participants, respectively. Of the 585 participants considered as refugees, 258 (44.1%) were actual refugees and 327 (55.9%) were the members of households who hosted these refugees. The Togolese seasonal economic migrants traveled mainly to the West African sub-region for work: Benin (2.7%), Nigeria (18.3%), Burkina Faso (22.4%), and Ghana (48.67%) (Fig. 2).

Of those who were interviewed on behalf of the household, with regard to household membership, 53.2% (740/1,391) were heads of households and 46.8% (651/1,391) were household members (Table 1).

**Table 1:** Sociodemographic characteristics of study participants

**Sociodemographic characteristics**	**Head of household** **(n = 740)**		**Household members** **(n = 651)**		**Total** **(N = 1391)**		***P-value***
	***n***	**(%)**	***n***	**(%)**	***N***	**(%)**	
Health district							< 0.001*
Kpendjal	262	35.4	27	4.1	289	20.8	
Oti	188	25.4	265	40.7	453	32.6	
Tandjouare	290	39.2	359	55.2	649	46.6	
Gender							0.491*
Female	366	49.5	334	51.3	700	50.3	
Male	374	50.5	317	48.7	691	49.7	
Age (years)							< 0.001**
0–5	0	0.0	176	27.1	203	14.6	
6–14	0	0.0	204	31.3	241	17.3	
15–49	624	84.7	245	37.6	805	57.9	
≥ 50	116	15.3	26	4.0	142	10.2	
Profession							< 0.001**
Shepherd	165	22.3	94	14.4	259	18.6	
Housewife	297	40.1	313	48.1	610	43.8	
Farmer	134	18.1	169	26.0	303	21.8	
Minor	22	3.0	0	0.0	22	1.6	
Unknown	78	10.5	36	5.5	114	8.2	
Other	44	6.0	39	6.0	83	6.0	
Type of migrant							< 0.001*
Economic migrant	225	30.4	212	32.6	437	31.4	
Nomad Peuhl	257	34.7	112	17.2	369	26.5	
Refugee	258	34.9	327	50.2	585	42.1	
Country of provenance							< 0.001**
Benin	31	4.2	6	0.9	37	2.7	
Burkina Faso	202	27.3	110	16.9	312	22.4	
Cote d'Ivoire	54	7.3	25	3.8	79	5.7	
Ghana	310	41.9	367	56.4	677	48.7	
Mali	1	0.1	0	0.0	1	0.1	
Niger	30	4.1	0	0.0	30	2.1	
Nigeria	112	15.1	143	22.0	255	18.3	

^*^Chi-square test; **Fisher test

### History of MDA, ITN use, LF complication, and ivermectin and albendazole distribution

The majority of participants (84.8%, 1180/1391) reported that they had never received MDA for LF. A significant proportion of participants, 38.2% (531/1391), did not have an insecticide-treated net (ITN), and only 48.7% (678/1391) reported having slept under ITN during the night before the survey. About 3.2% (44/1391) reported a history of LF complication including hydrocele, elephantiasis, and lymphedema. FTS positivity was statistically different according to LF complications (*P* < 0.001) and migrant group (*P* < 0.001) (Table 2).

**Table 2:** History of MDA, ITN use, LF complication, and ivermectin and albendazole distribution by FTS results, Togo

	**Filariasis test strips (FTS)**			***P***
	**Negative**	**Positive**	**Total**	
Mass drug administration for LF*, *n* (%)				< 0.001**
No	1155 (97.9)	25 (2.1)	1180	
Yes	170 (83.7)	33 (16.3)	203	
Unknown	8 (100.0)	0 (0.0)	8	
ITN availability*,* n* (%)				0.354*
No	505 (95.1)	26 (4.9)	531	
Yes	828 (96.3)	32 (3.7)	860	
ITN use* during the night before the survey, * n* (%)				0.657**
No	667 (95.6)	31 (4.4)	698	
Yes	651 (96.0)	27 (4.0)	678	
Unknown	15 (100.0)	0 (0.0)	15	
History of LF complications,* n* (%)				< 0.001*
No	1297 (96.3)	50 (3.7)	1347	
Yes	36 (81.8)	8 (18.2)	44	
Type of LF complication (n = 44),* n* (%)				0.006**
Elephantiasis/ lymphedema	11 (100.0)	0 (0.0)	11	
Hydrocele/ elephantiasis/ lymphedema	2 (66.7)	1 (33.3)	3	
Hydrocele	19 (90.5)	2 (9.5)	21	
Unknown	4 (44.4)	5 (55.6)	9	
Ever ivermectin (for onchocerciasis) and/or albendazole (for STH) distribution,* n* (%)				< 0.001**
No	1322 (100.0)	0 (0.0)	1322	
Yes	11 (15.9)	58 (84.1)	69	
Type of migrant,* n* (%)				< 0.001*
Economic migrant	432 (98.9)	5 (1.1)	437	
Nomad Peuhl	325 (88.1)	44 (11.9)	369	
Refugee	576 (98.5)	9 (1.5)	585	

(*) Self-report based on recall; *Chi-square test; **Fisher test

*MDA* mass drug administration, *ITN* insecticide-treated net, *LF* lymphatic filariasis, *STH* soil-transmitted helminths

### Prevalence of lymphatic filariasis

**Fig. 3:**
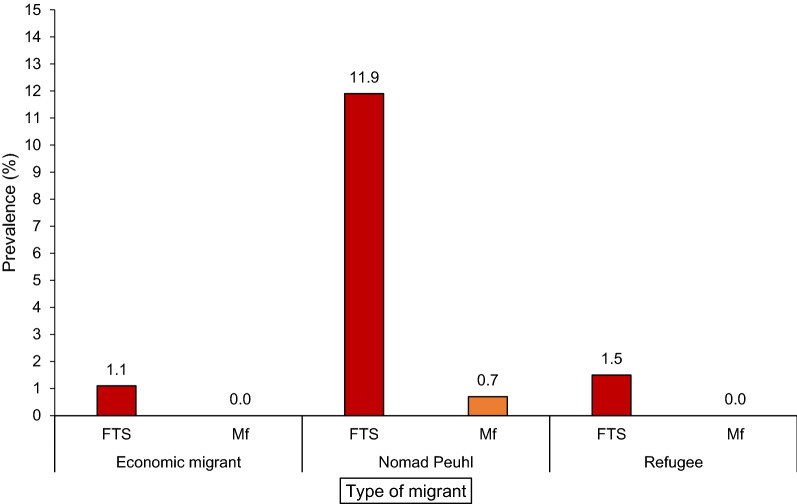
Prevalence of CFA and microfilaremia by migrant type

The prevalence of circulating filarial antigen (CFA) by FTS was 4.2% (58/1391), 95% CI [3.2–5.4] overall, but it varied across the migrant groups: 11.9% (95% CI = [8.6–15.2]) in the nomadic Peuhl group, 1.5% (95% CI = [0.5–2.5]) in refugees and 1.1% (95% CI [0.1–2.1]) among economic migrants (*P* < 0.001). Among the five economic migrants with a positive FTS result, four traveled to Côte d’Ivoire and one traveled to Burkina Faso for work. Only one case (0.07%) from the nomadic Peuhl group was confirmed by nocturnal microfilaremia (Fig. 3).

The positive case was a male member of a household, aged 15–49 years, found in Sadori village in the Oti district. He is a nomadic Peuhl from Burkina Faso. Investigations among other household members did not reveal any other microfilaremia-positive case.

## Discussion

This study examined the risk that three migrant groups in the north of Togo pose for the reintroduction of LF to the country. The prevalence of circulating filarial antigen was low among Togolese who travel to other countries for seasonal work and among Ghanaian refugees residing in Togo, but the prevalence of CFA was 11.9% among nomadic Peuhls, suggesting that this group could serve as a reservoir of infection for the reintroduction of LF to Togo. These findings underscore the importance of implementing post-validation surveillance activities as soon as elimination of LF as a public health problem is achieved.

In 2017, the WHO recognized Togo as the first sub-Saharan country to have eliminated LF as a public health problem, based on the excellent results of the surveillance activities conducted during the 5-year post-MDA period. Despite the absence of transmission among the autochthonous population in Togo, ongoing intensive surveillance for LF is important and necessary to preserve this success, especially in the post-validation period. Post-MDA surveillance is recommended by the WHO [12], but the specific strategies must be identified and proposed to the countries that have achieved validation to be implemented in the framework of post-validation surveillance (PVS). This is particularly critical for countries such as Togo, which is neighbored by countries still endemic for LF with a potential risk of a reintroduction.

To address this issue, innovative approaches must be developed by the countries to reach the LF elimination endgame, especially in the absence of PVS guidelines. Therefore, Togo tried to implement some strategies to document the absence of *W. bancrofti*; the migrant groups in the three previous endemic districts in Savanes region in Togo need to be documented and examined to detect the risk of reemergence of LF disease related to these migrant groups. The approaches to contain this risk exist and could be efficient. Indeed, some countries such as China, Japan, Korea, Thailand, Solomon Islands, Sri Lanka, Brazil, Malaysia, Costa Rica, Suriname, and Trinidad and Tobago, with different epidemiological contexts, have demonstrated that LF transmission can be permanently stopped by improving monitoring, follow-up, and evaluation, improving the drug distribution system, improving social mobilization approaches, linking LF to other disease interventions, and increasing human health worker capacity [13].

Migrants have been recognized to have the potential to threaten the achievements of the LF elimination programs in some areas of the world [1]. For example, Malaysia is not endemic for *W. bancrofti*, but to prevent the introduction of LF, the country has adopted a strategy to follow up the foreign labor serving the low- and semi-skilled sectors of the economy, a labor force comprised primarily of people coming from LF-endemic countries. In a study conducted among 484 migrant workers from six countries, both *brugian* and *bancroftian* filariasis were investigated using rapid tests based on detection of specific IgG4 antibodies against *BmR1 (Brugia Rapid)* and *BmSXP* recombinant antigens. A prevalence of 10% was found for BmR1 and 33% for BmSXP [14]. These estimates were higher than the proportion of FTS-positive cases reported among economic migrants in our study (1.1%). This gap could mainly be explained by the difference in the number of migrants in the two studies; also, unlike the Malaysian study that looked for antibodies, in our study we were looking for antigens.

In addition, LF is a complex disease, usually silent in the early stages, making untreated cases of *W. bancrofti* in the community a reservoir of the disease. The delay between LF infection and the onset of morbidity symptoms can be 10 years or more, hence the critical importance of maintaining surveillance activities after elimination [6, 15]. It has also been noted that the distribution of LF in the world has been attributed to migration [16, 17], and the movement of infected people to non-endemic areas may introduce infection into new areas [18] or reintroduce active transmission to previously endemic areas. Each year, in some region of the world, about 2.0–2.5% of the endemic rural population migrates, increasing the risk of introducing infection or prolonging the "residual infection" phase in their predominantly urban destinations. Migrants have the potential to counteract the results of control/elimination programs in areas where the vector-parasite combination is conducive to transmission, especially in cross-border areas [19], although in Togo, the vector evaluation showed an absence of infestation in mosquitoes collected in 2016, 6 years after the cessation of MDA [9].

Cross-border migration also threatens to increase LF. One of the best known examples is the Thai-Myanmar border. Border areas are endemic for LF and pose a challenge to LF elimination efforts in Thailand. Microfilaremia rates of up to 6.0%, antigen prevalence between 22.0% and 36.8% and antibody prevalence of 54% have been reported in border areas of Thailand [20, 21]. The elimination of LF in this area is hampered by the influx of migrants from Myanmar. The prevalence of MF among these migrants is between 4.4% and 8.0%, the prevalence of antigen between 10.0% and 24%, and an antibody prevalence of 42% has been reported [22, 23]. In Togo, passive surveillance carried out in 2010, which had focused on border areas, and mapping confirm that the risk of importation of LF is likely low for border areas [5], and another study confirmed this low cross-border risk [10]. Border surveillance concerns the inhabitants on both sides of the borders under consideration, including possibly migrants. However, typically only sick or suffering people who go to health centers for consultation are considered for infections; asymptomatic LF patients may not be identified. Moreover, the border surveillance only takes into account people who are legally crossing the borders; thus, some migrants who do not pass through the official roads where the border surveillance is carried out may escape the border surveillance.

Worldwide, millions of people from endemic areas or countries migrate to non-endemic areas within or outside their countries. Four categories of migration have implications for LF elimination efforts. These are (i) migration from endemic to non-endemic areas, (ii) migration from endemic rural to endemic urban areas, (iii) migration from endemic areas to areas that have successfully controlled/eliminated LF, and (iv) cross-border migration [19]. The group of nomadic Peuhls who come from and to neighboring endemic countries while crossing Togo, and who represent a high risk of reintroduction of LF in Togo, constitute a fifth category: (v) cross-country migration.

In our study, we assumed that three groups could contribute to an increased risk of LF resurgence for Togo. The northern part of the country was previously the most endemic area for LF according to the baseline prevalence of microfilaraemia found. In addition, this area borders southern Burkina Faso, which is highly endemic to FL with a migratory flow between the two countries in this area. It is also through these northern districts that nomadic Peuhls repeatedly pass to reach Ghana from Burkina Faso. In addition, the communities of these districts according to their customs must return to their native countries for the end of year celebrations and funeral ceremonies. These factors together make the north an area where the risk of resurgence is high. There is also a risk associated with individual migrants coming from other countries to various locations in Togo, but it is the strong migratory movement in the north in a given period that elevates the risk in the north.

We found that for Togolese economic migrants who work in still-endemic countries and who return to Togo at specific times of the year, the prevalence of LF is relatively low (1.1%); the same is true for refugees from endemic countries (1.5%). The low prevalence in the latter group may be explained in part by ongoing annual or twice-yearly MDA with ivermectin for onchocerciasis and albendazole in school-age children for STH in the districts included in this survey. The only group that constitutes a real risk of LF resurgence and warrants close follow-up is the group of nomadic Peuhls who cross the country coming from Benin, Ghana, and Burkina Faso. In this group, the prevalence of CFA was 11.9%, and the one MF-positive case found was in the group. About 85% people in this group surveyed are naïve to treatment. Given that they do not take preventive chemotherapy and that LF is silent in the early stages, these nomads continue to be a parasite reservoir, thus increasing the risk of disease resurgence. This result confirms those already obtained in Togo in 2006 before the cessation of mass treatment, when one of the two MF-positive cases detected by the passive surveillance system was a member of the nomadic Peuhl group crossing borders to other LF-endemic areas in Benin, Ghana, and Burkina Faso. This patient could not be located for follow-up, preventing a local investigation [6]. Nevertheless, ongoing ivermectin and albendazole MDA in these districts may be helping prevent the spread of any LF infection imported by these nomads. The risk of reintroduction through this group will increase when MDA for onchocerciasis and STH eventually stop in the absence of specific control or surveillance activities targeting this population.

The implementation of a strategy specifically adapted to the nomadic Peuhl group to contain the potential risk of LF resurgence should focus on two points: (i) directing and intensifying awareness about the use of impregnated mosquito nets by this group; (ii) involving veterinary posts, that already work closely with nomadic Peuhls at particular times, in providing treatment for LF.

In our study, a minority of participants (48.7%) reported having slept under ITN the night before the survey. Efforts should be made to raise awareness about the importance of using ITNs as they will be the only tool available to combat LF once MDA with ivermectin for onchocerciasis and albendazole for STH ends.

Little is known about migrant groups in the context of LF in West Africa, and to our knowledge this study is the first that has assessed migrant groups to determine the potential risk of LF reemergence after its elimination.

Our study has some limitations. Not taking into account all regions of Togo may be a limitation; indeed, other regions or districts of Togo may be subject to migratory movements that will need to be documented in future studies. Another limitation may be the relatively small sample size.

Since LF has been eliminated in Togo, financial support for LF-related tasks has decreased, and the program to conduct surveillance and morbidity management cannot be carried out effectively. One of the potential challenges of the Togo LF program now is to develop and maintain a long-term sustainable surveillance system that is integrated into the existing health infrastructure.

## Conclusion

This study, which included 1391 nomadic Peuhls, refugees, and economic migrants, showed a high prevalence of circulating filariasis antigen among nomadic Peuhls (11.9%) and a lower prevalence among refugees (1.5%) and economic migrants (1.1%). Only one of 58 positive cases by FTS was confirmed by microfilaremia in the nomadic Peuhl group.

Consequently, this study suggests that while most migrant groups do not pose a significant risk for LF reemergence after stopping MDA in Togo, nomadic Peuhls do pose a risk, and migration to and from neighboring countries that are still endemic will continue. It is therefore important to monitor this specific group as a post-validation surveillance activity to promptly detect any LF cluster in the country until the WHO proposes an official framework for post-validation LF surveillance.

## Data Availability

The datasets used and analyzed during this study are available from the corresponding author upon reasonable request.

## Abbreviations

CFA: Circulating filariasis antigen
ITN: Insecticide-treated net
LF: Lymphatic filariasis
FTS: Filariasis test strip
MDA: Mass drug administration
NPELF: National Program for Elimination of Lymphatic Filariasis
PTS: Post-treatment surveillance
PVS: Post-validation surveillance
TAS: Transmission assessment survey
WHO: World Health Organization
